# Supplementary material for: Natural variations at the Stay-Green gene promoter control lifespan and yield in rice cultivars
Source: Nat Commun. 2020 Jun 4;11:2819. doi: 10.1038/s41467-020-16573-2 (PMC7272468; doi:10.1038/s41467-020-16573-2)
Supplement: Supplementary file 2 — Supporting information [file 41467_2020_16573_MOESM2_ESM.pdf]

Supplementary Information

Supplementary Figures

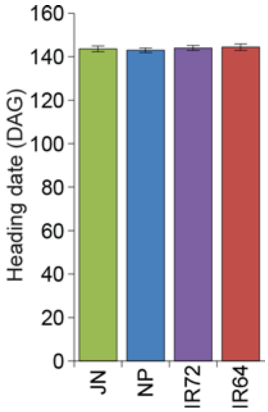

Supplementary Figure 1. Heading dates of two *japonica* (JN, Junam; NP, Nampyeong) and two *indica* (IR72, IR64) rice plants. DAG; days after germination. Values are means ± SE (n = 10).

Source data of Supplementary Figure 1 are provided as a Source Data file.

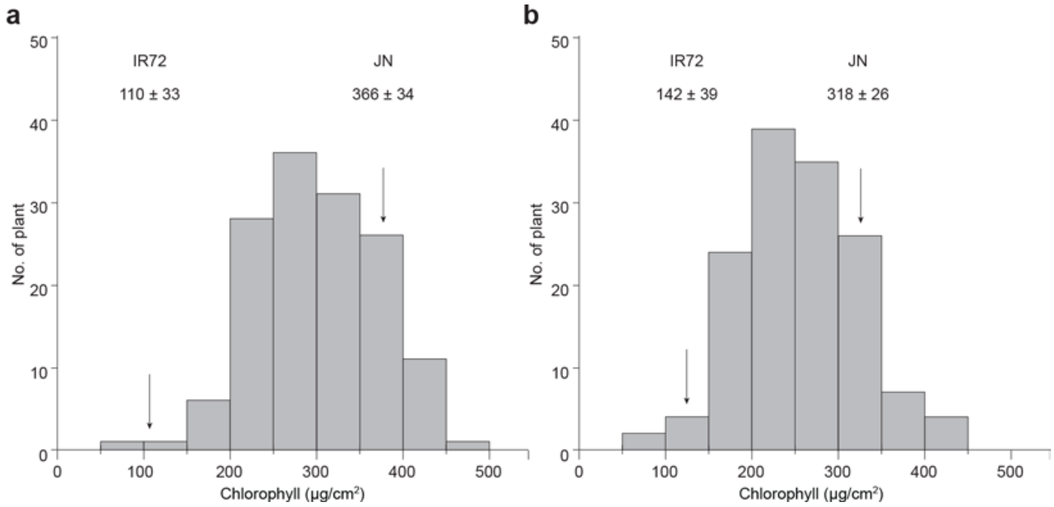

Supplementary Figure 2. Frequency distributions of chlorophyll content associated with leaf senescence in 141 $F_{2:3}$ populations derived from IR72 and JN. Chlorophyll content of flag leaves (**a**) and second upper leaves (**b**). Chlorophyll contents of flag leaves and second upper leaves were measured at 6 weeks after heading. Mean of chlorophyll content was used ($n \geq 6$). Source data of Supplementary Figure 2a and b are provided as a Source Data file.

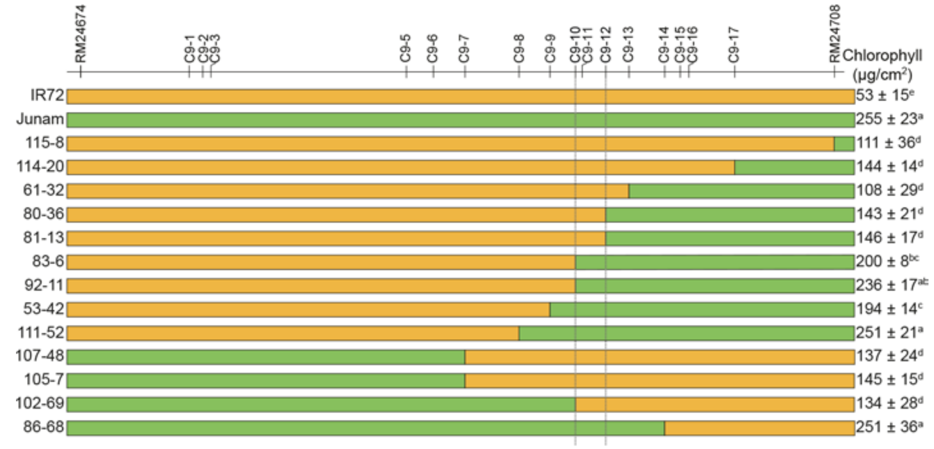

Supplementary Figure 3. Graphical genotypes of critical recombinants and their chlorophyll levels in flag leaves used for fine-mapping. Genotyping of progeny for the defined the locus to 26 kb region flanked by markers C-10 and C12. Chlorophyll levels in flag leaves are shown for recombinants BC_5F_2 plants and parental lines at 6 weeks after flowering. Orange bars represent *indica*-type; green bars represent *japonica*-type. Chlorophyll levels are shown as means ± SE (n = 10). Different lowercase letters indicate significant differences (P < 0.05 by Student's *t*-test). Source data of Supplementary Figure 3 are provided as a Source Data file.

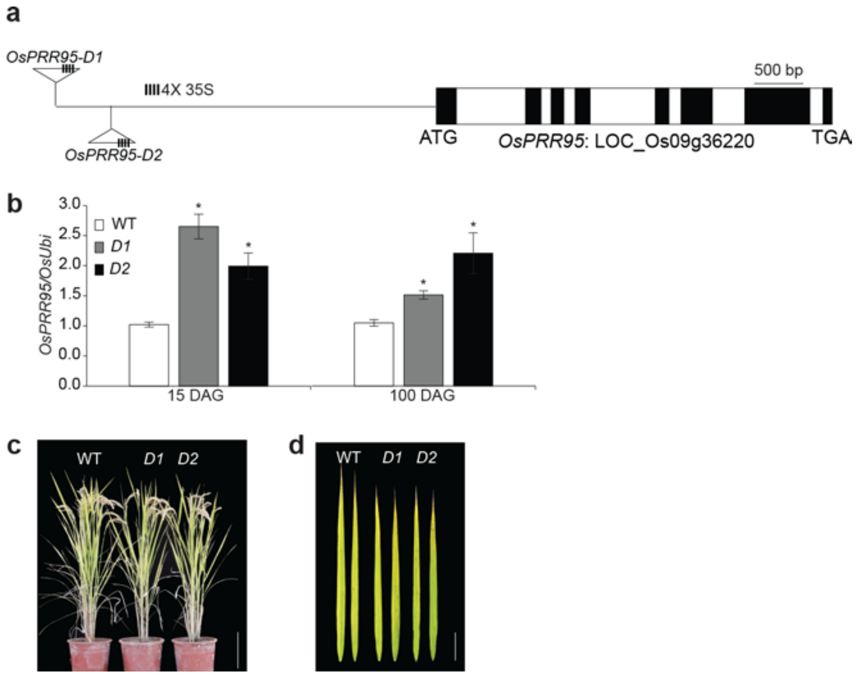

Supplementary Figure 4. Leaf senescence phenotype of activation tagging mutants of *OsPRR95*. **a**, Schematic diagram of T-DNA insertion sites in *OsPRR95-D1* and *OsPRR95-D2* mutants. 4× 35S denotes 4 copies of *35S* enhancer. **b**, qRT-PCR analysis of *OsPRR95* expression using RNA from 15 and 100 DAG leaves from WT and two activation tagging mutants. DAG: Days after germination. Significant differences between WT and mutant plants were determined using Student's *t*-test`, and indicated with an asterisk (*$P < 0.05$). Data are shown as means ± SE ($n = 3$). **c**, Senescence phenotypes of WT and mutants grown in the paddy field. Bar, 20 cm. **d**, Flag leaves from WT and mutants before harvest. Bar, 5 cm. Source data of Supplementary Figure 4b are provided as a Source Data file.

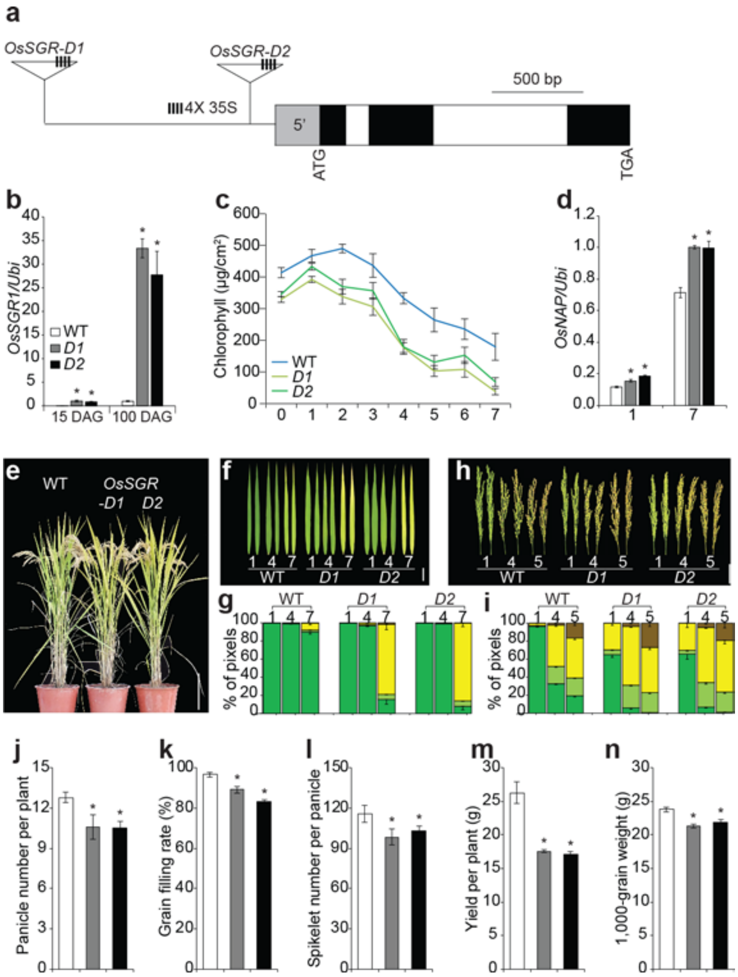

Supplementary Figure 5. Activation tagging mutants of *OsSGR*. **a**, Schematic diagram of T-DNA insertion sites in *OsSGR-D1* and *OsSGR-D2* mutants. **b**, qRT-PCR analysis of *OsSGR* expression using RNA from 15 and 100 DAG leaves from WT and two activation tagging mutants. DAG: Days after germination. Asterisks (*) indicate significant differences between WT and mutants (*$P < 0.05$ by Student's *t*-test). **c**, Temporal changes of chlorophyll levels in flag leaves of WT and mutants from 0 to 7 weeks after heading. Values are means ± SE ($n = 5$). **d**, Expression analysis of *OsNAP* in flag leaves at 1 and 7 weeks after heading. Data are means ± SE ($n = 3$). *$P < 0.05$ by Student's *t*-test. **e**, Representative senescence phenotypes of activation tagging mutants of *OsSGR* in *japonica* subspecies grown in natural field conditions. Bar, 20 cm. Color changes (**f**, **h**) and their quantified values (**g**, **i**) of the flag leaves (**f**, **g**) and panicles (**h**, **i**) of WT and the activation tagging mutants during seasonal senescence. Bars, 5 cm. In colourimetric assay, the same scale is used as in Fig. 1. Values are means ±

SE ($n \geq 4$). Number of panicles per plant (**j**), grain filling rate (**k**), number of spikelet per panicle (**l**), total grain yields per plant (**m**), and 1,000-seed weight (**n**). Values are given as the mean ± SE ($n \geq 5$). *$P < 0.05$ by Student's *t*-test. Source data of Supplementary Figure 5b-d, g and i-n are provided as a Source Data file.

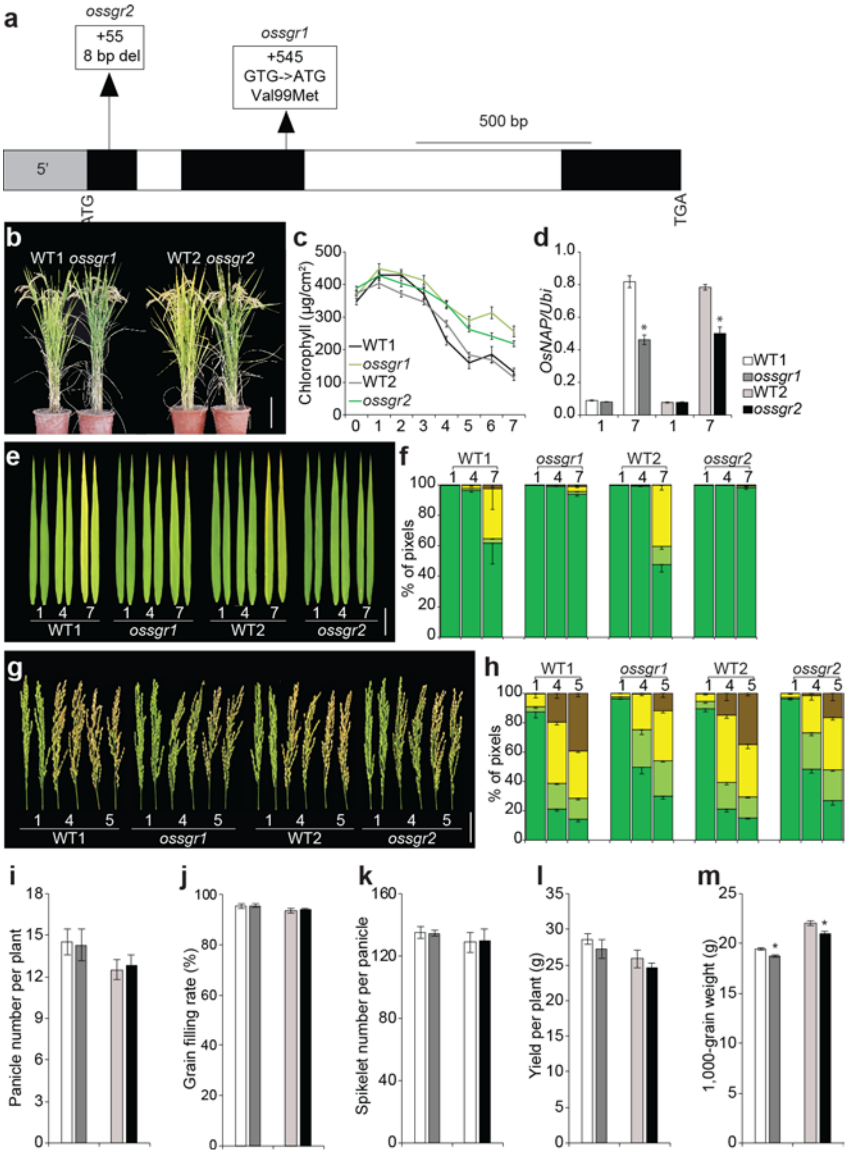

Supplementary Figure 6. Phenotypes of *ossgr* mutants in *japonica* subspecies. a. Schematic representation of *OsSGR* structure and the mutation positions in *japonica* subspecies**.** Black and white boxes represent exons and introns, respectively. Gray boxes indicate 5’ untranslated region. **b,** Representative senescence phenotypes of *ossgr* mutants in two *japonica* type cultivars. WT1, Hwacheong; WT2, Nipponbare. Bar, 20 cm. **c**, Temporal changes of chlorophyll levels in flag leaves of WTs and mutants from 0 to 7 weeks after heading. Data are means (± SE) of five plants. **d**, Transcript levels of *OsNAP* in flag leaves at 1 and 7 weeks after heading. Data are means ± SE (n = 3). *P < 0.05 by Student’s t-test. Color changes (**e**) and quantification of color (**f**) of flag leaves from

1, 4 and 5 weeks after heading. Color change (**g**) and quantification of colors (**h**) of panicles from 1, 4 and 5 weeks after heading. Values are means ± SE ($n \geq 4$). In colourimetric assay, the same scale is used as in Fig. 1. Number of panicles per plant (**i**), grain filling rate (**j**), number of spikelet per panicle (**k**), total grain yields per plant (**l**), and 1,000-seed weight (**m**). Values are given as the mean ± SE ($n \geq 5$). *$P < 0.05$ by Student's *t*-test. Source data of Supplementary Figure 6c, d, f and h-m are provided as a Source Data file.

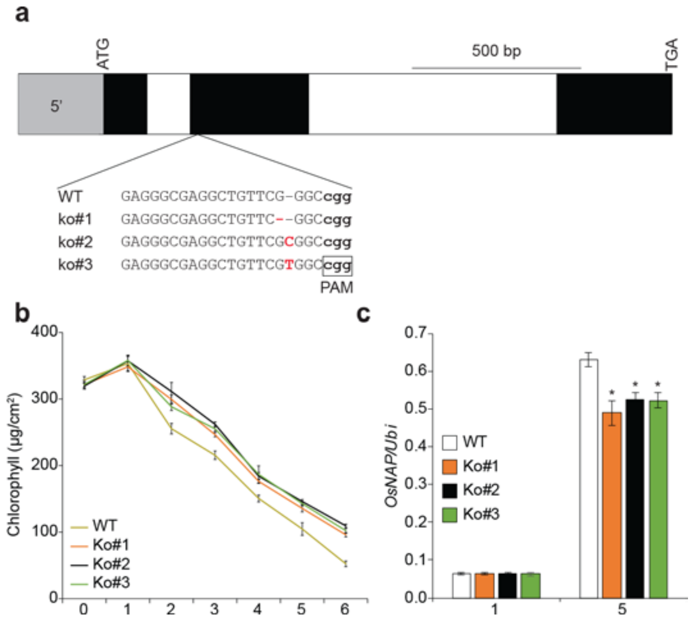

Supplementary Figure 7. Generation of *ossgr* mutants in *indica* rice variety Kasalath via CRISPR/Cas9 genome editing. a, Schematic representation of *OsSGR* structure and the *ossgr* mutation position induced by CRISPR/Cas9 editing in the T0 generation transgenic Kasalath plants. Sequencing results of mutant alleles are aligned to the WT sequence. The indels are shown in red letters or dashes. The PAM site is highlighted in bold face fonts. **b**, Temporal changes of chlorophyll levels in flag leaves of WT and *ossgr* mutants from 0 to 6 weeks after heading. Values are means ± SE (n = 6). **c**, Expression levels of *OsNAP* in flag leaves at 1 and 5 weeks after heading. Data are means ± SE (n = 3). *P < 0.05 by Student's t-test. Source data of Supplementary Figure 7b and c are provided as a Source Data file.

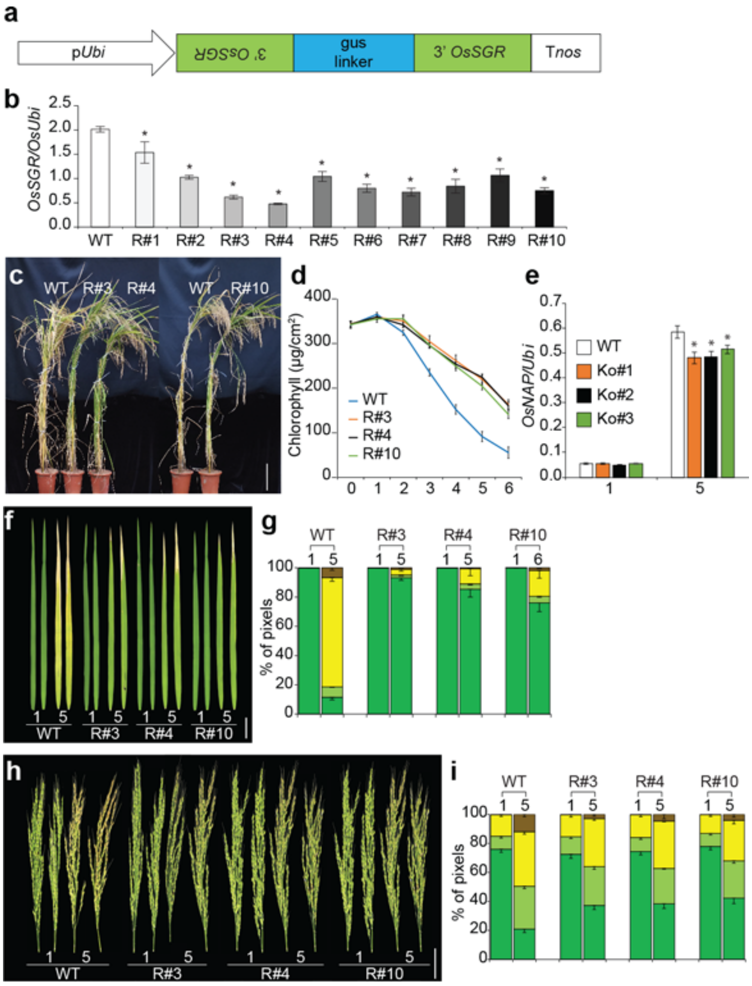

Supplementary Figure 8. Phenotypes of *OsSGR* RNAi lines in *indica* (Kasalath) subspecies. a, Schematic representation of the vector expressing *OsSGR* under control of maize *ubiquitin* promoter (p*Ubi*) and *nopaline synthase* terminator (T*nos*). **b**, qRT-PCR analysis of *OsSGR* expression using RNA from 100 DAG leaves from WT and transgenic plants. Significant differences between WT and transgenic plants are indicated with an asterisk (*$P < 0.05$ by Student's *t*-test). **c**, Representative senescence phenotypes of WT and transgenic plants (R#3, R#4 and R#10). Bar, 20 cm. **d**, Temporal changes of chlorophyll levels in flag leaves of WT and transgenic plants. Values are means ± SE (n = 6). **e**, Expression analysis of *OsNAP* in flag leaves at 1 and 5 weeks after heading. Data are means ± SE (n = 3). *$P < 0.05$ by Student's *t*-test. Color changes (**f**, **h**) and quantification (**g**, **i**) of the flag leaves (**f**, **g**) and panicles (**h**, **i**) of WT and the RNAi transgenic plants during seasonal senescence (1

and 5 weeks after heading). Bars, 5 cm. Values are means ± SE ($n \geq 4$). In colourimetric assay, the same scale is used as in Fig. 1. Source data of Supplementary Figure 8b, d, e, g and i are provided as a Source Data file.

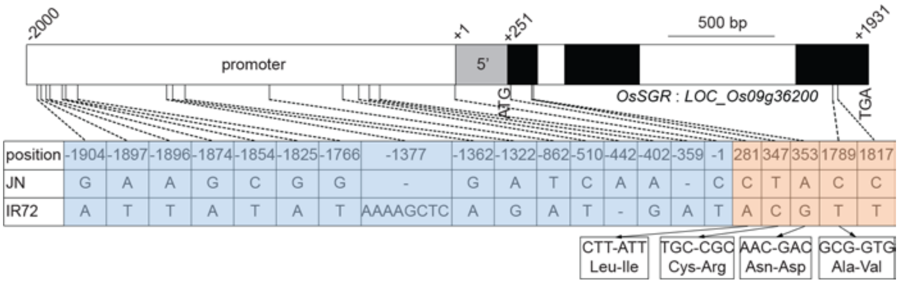

Supplementary Figure 9. Allelic variations in the *OsSGR* gene. Schematic representation of genetic variations in *OsSGR* in *japonica* variety (JN) and *indica* variety (IR72). There are 4 amino acid changes in the coding region (shaded in orange), and 13 SNPs and 3 indels in the promoter region (blue).

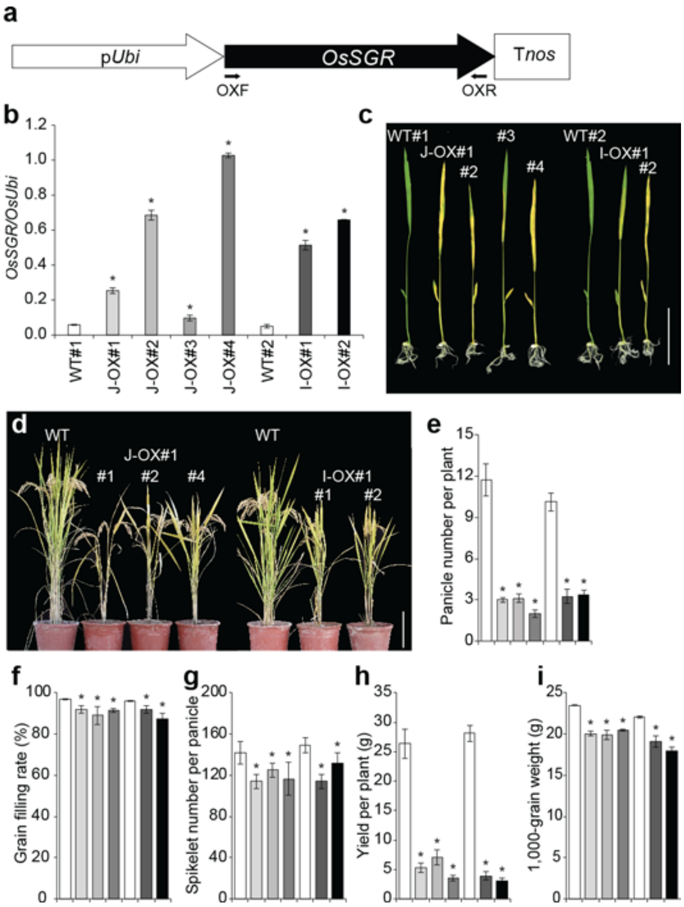

Supplementary Figure 10. Generation and characterization of two types of *OsSGR* overexpressing transgenic plants. a, Schematic representation of the vector expressing *OsSGR* under the control of maize *ubiquitin* promoter (p*Ubi*) and *nopaline synthase* terminator (T*nos*). **b**, qRT-PCR analysis of *OsSGR* in WT (JN; *japonica*) and *OsSGR* overexpressing (OX) transgenic plants, using RNA from 7-days old seedling shoots. J-OX: *japonica* (JN)-type *OsSGR* overexpressing transgenics: I-OX: *indica* (IR72)-type *OsSGR* overexpressing transgenics. Values are means ± SE (n = 3). Significant differences between WT and transgenic plants are indicated with an asterisk (*P < 0.05 by Student's t-test). **c**, Phenotype of 7-days old transgenic seedling plants. Bar, 5 cm. **d**, Representative senescence phenotypes of transgenic plants. Bar, 20 cm. Number of panicles per plant (**e**), grain filling rate (**f**), number of spikelet per panicle (**g**), total grain yields per plant (**h**), and 1,000-seed weight (**i**). Values are given as the mean ± SE ($n \geq 5$). *P < 0.05 by Student's t-test. Source data of Supplementary Figure 10b and e-i are provided as a Source Data file.

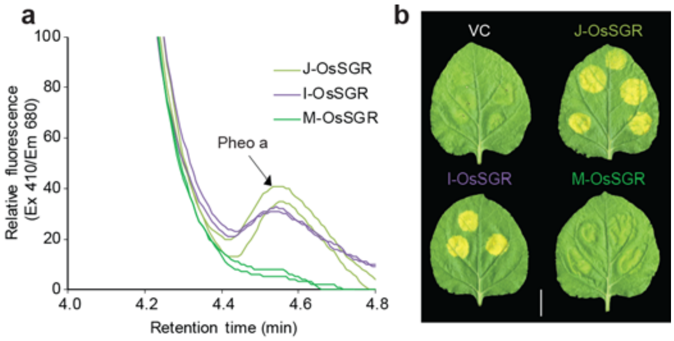

Supplementary Figure 11. Analysis of biochemical activity of three types of OsSGR. a, Comparison of Mg^{++}-dechelatase activities of *japonica* (JN) - (J-OsSGR: dark green), *indica* (IR72) - (I-OsSGR: purple), and *ossgr* (M-OsSGR: green) type proteins. After incubation of chlorophyll a with OsSGR, the level of pheophytin a (Pheo a), a product of Mg^{++}-dechelatase were determined by HPLC. Pigments were detected at 610 nm emission. **b**, Tobacco infiltration assay using two types of OsSGR. Infiltration was performed by infecting the prepared *Agrobacterium* containing using OsSGR into 4 weeks old *Nicotiana benthamiana* leaves. Photos were taken 4 days after Infiltration.VC; vector control. Bar, 5 cm. Source data of Supplementary Figure 11a are provided as a Source Data file.

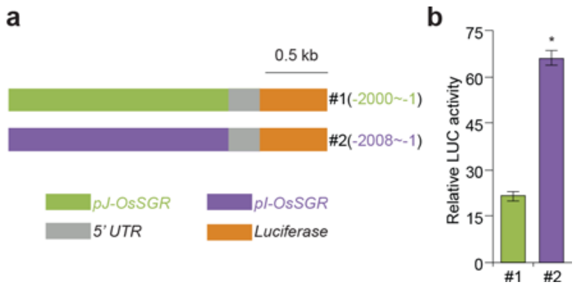

Supplementary Figure 12. Promoter analysis of *OsSGR* using transient systems. a, Diagrams of the *OsSGR* promoter and 5’ UTR fused to the Luciferse genes. Green and purple colors indicate *japonica* (JN)-type and *indica* (IR72)-type *OsSGR* promoter, respectively. Grey boxes: 5’ UTR; Orange boxes: luciferase genes. **b**, Transient assay using rice Oc cell protoplasts to test *OsSGR* promoter activity. Values are means ± SE (n = 4). *P < 0.05 by Student’s t-test. Source data of Supplementary Figure 12b are provided as a Source Data file.

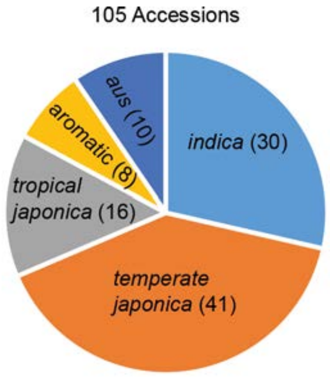

Supplementary Figure 13. Number of accessions used in this study. Accessions include the five groups of *O. sativa* such as *indica*, *temperate japonica*, *tropical japonica*, *aromatic* and *aus*.

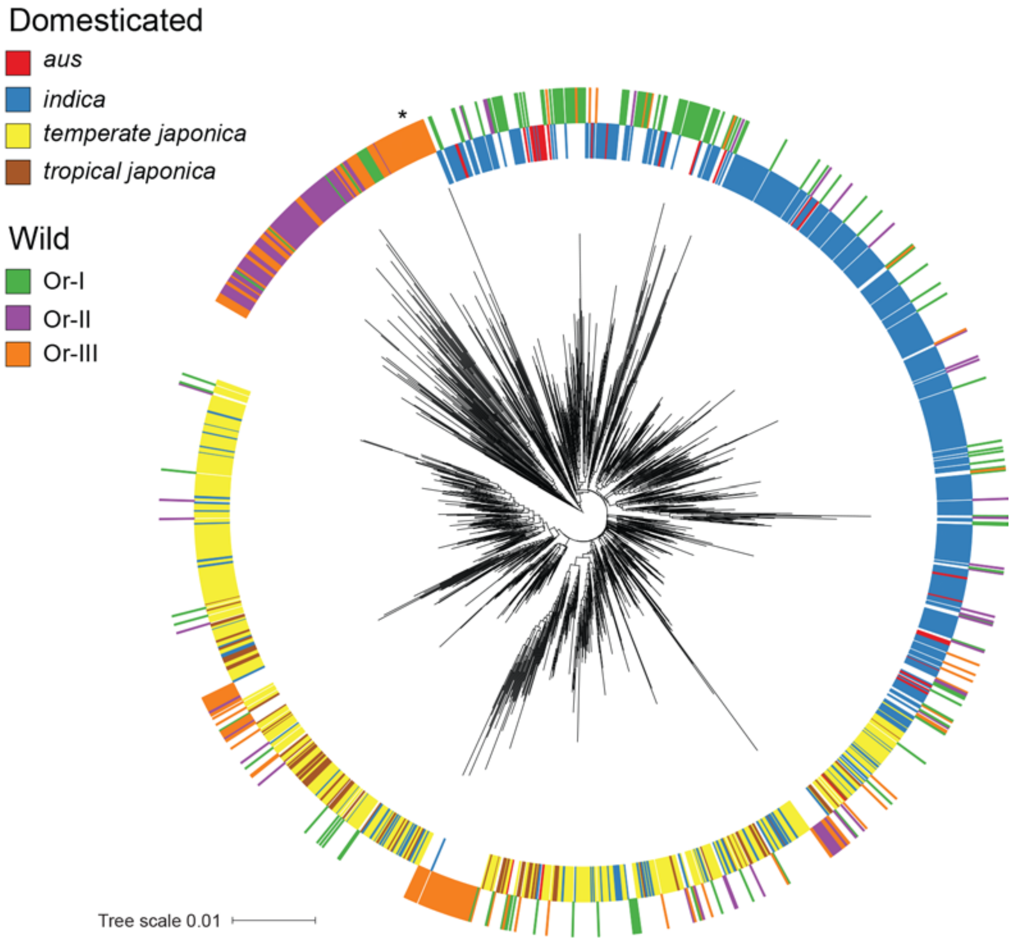

Supplementary Figure 14. Neighbor-joining tree for 10 kb upstream and downstream of *OsSGR* gene region from 1477 domesticated and wild rice samples. Inner circles of colors represent domesticated rice: red, *aus*; blue, *indica*; yellow, *temperate japonica*; and brown, *tropical japonica*. Outer circles of colors represent wild rice as designated by Huang et al. (2012): green, Or-I/*O. nivara*-like; purple, Or-II; and orange, Or-III/*O. rufipogon*-like. Or-II has been shown to be the most genetically distinct wild rice group by Huang et al. (2012). Star represents a majority Or-III group that is sister to all domesticated and wild rices.

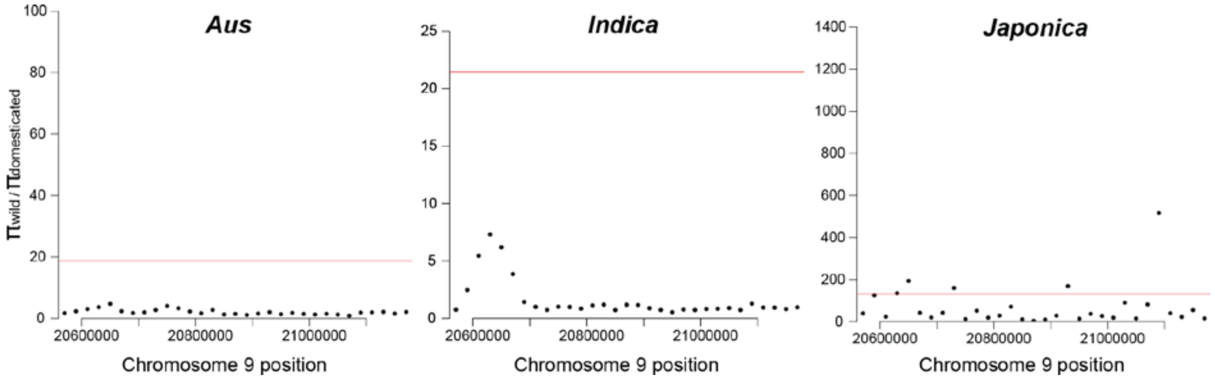

Supplementary Figure 15. Polymorphism of wild to domesticated rice (π_W/π_D) statistics around the *OsSGR* gene region (chr9:20,868,846 ~ 20,871,077) for the domesticated rices. π_W/π_D values were estimated using a 20 kb sliding window. The top 1% threshold is shown with a red line.

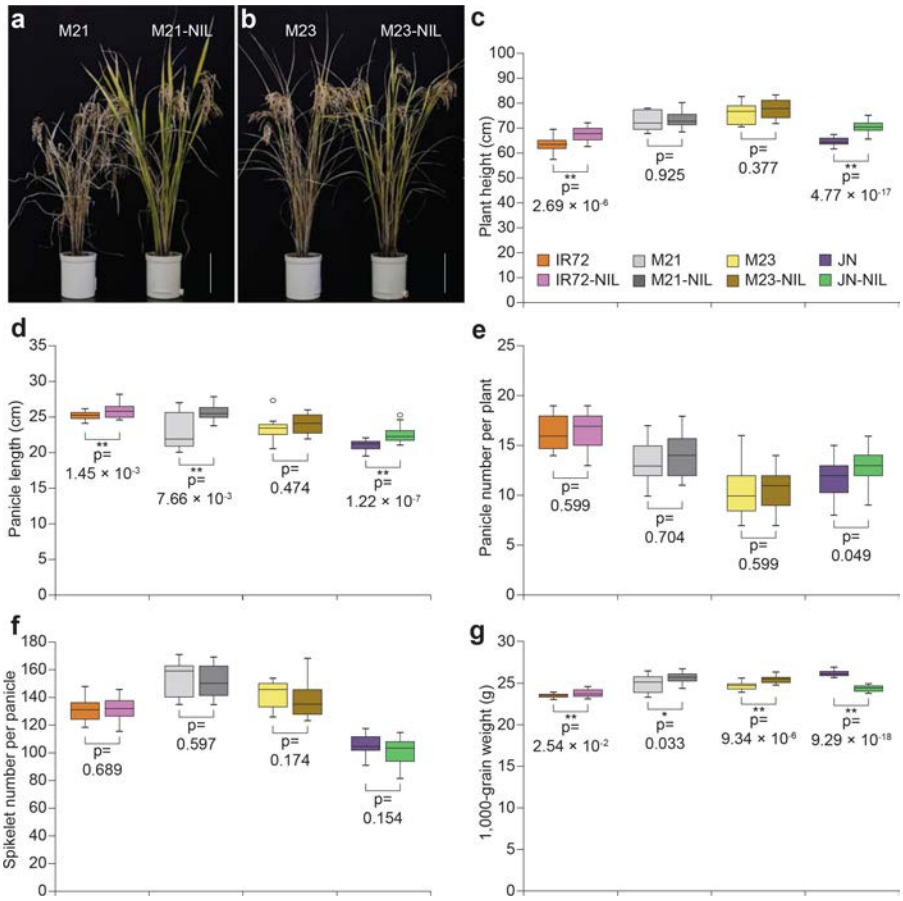

Supplementary Figure 16. Agronomic traits of NILs grown in field. a, Representative senescence phenotypes of Milyang21 (M21; *indica*) and M21-NIL harboring *japonica OsSGR* grown in natural fields. Bar, 20 cm. **b**, Senescence phenotypes of Milyang23 (M23; *indica*) and M23-NIL harboring *japonica OsSGR* grown in seasonal growth condition. Bar, 20 cm. Plant height (**c**), panicle length (**d**), Number of panicles per plant (**e**), Number of spikelets per panicle (**f**) and 1,000-seed weight (**g**). Values are given as the mean ± SE ($n \geq 10$). *P < 0.05, **P < 0.1 by Student's *t*-test. In the box plots, the center value is the median and the bottom and top edges of the boxes display 1.5 times the interquartile range. Source data of Supplementary Figure 16c-g are provided as a Source Data file.

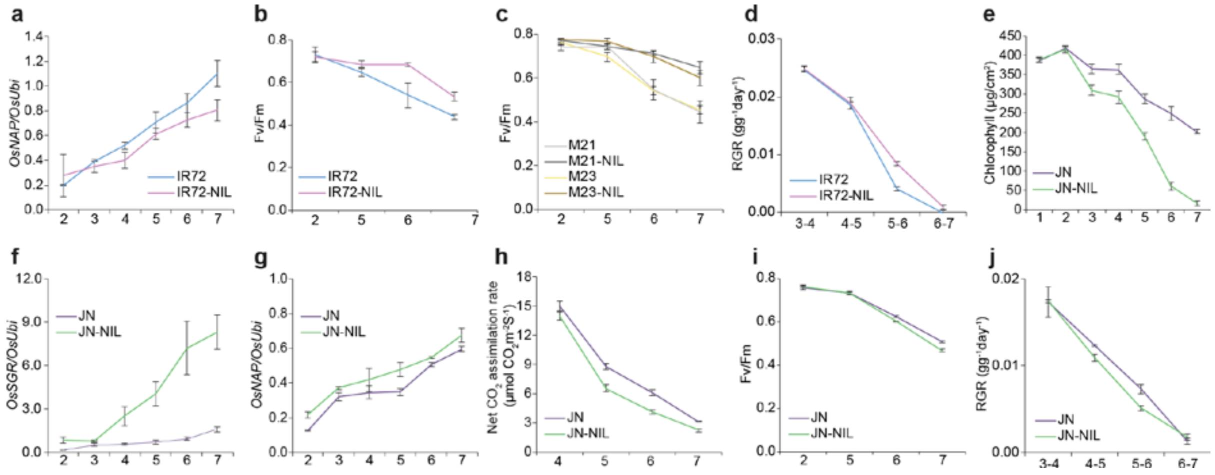

Supplementary Figure 17. Characterization of NILs grown in field. a, Transcript levels of *OsNAP* in the flag leaves from NILs from 2 to 7 weeks after heading. Data are means ± SE (*n* = 3). **b**, Temporal patterns of Fv/Fm values in flag leaves of IR72 and IR72-NIL during the grain-filling stage (2, 5, 6, and 7 weeks after heading). **c**, Analysis of Fv/Fm ratios in flag leaves of M21, M21-NIL, M23 and M23-NIL. Values are given as the mean ± SE (*n* = 5). **d**, Relative growth rate (RGR) of IR72 and IT72-NIL grown in the field. Time intervals are 3-4, 4-5, 5-6, and 6-7 weeks after heading. Values are given as the mean ± SE (*n* = 3). **e**, Temporal changes of chlorophyll levels of flag leaves from 1 to 7 weeks after heading in JN and JN-NIL. Data are means ± SE (*n* = 8). Expression patterns of *OsSGR* (**f**) and *OsNAP* (**g**) in the flag leaves from JN and JN-NIL from 2 to 7 weeks after heading. Data are means ± SE (*n* = 3). **h**, The analysis of the net CO_2 assimilation rate in flag leaves of JN and JN-NIL during the grain-filling stage (4, 5, 6 and 7 weeks after heading). Data are means ± SE (*n* = 5). **i**, Temporal patterns of Fv/Fm ratios in flag leaves of JN and JN-NIL during the grain-filling stage (2, 5, 6, and 7 weeks after heading). Values are given as the mean ± SE (*n* = 5). **j**, RGR of JN and JN-NIL grown in the field. Values are given as the mean ± SE (*n* = 3). Source data of Supplementary Figure 17a-j are provided as a Source Data file.

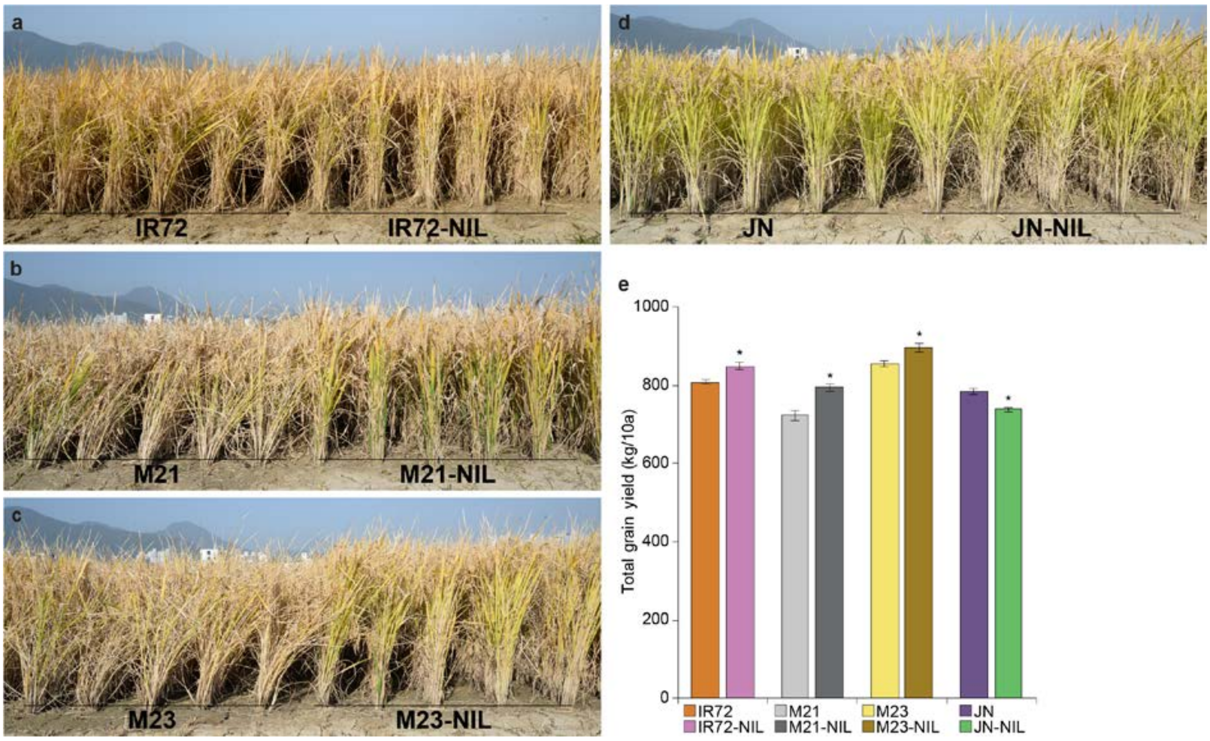

Supplementary Figure 18. Senescence phenotypes of NILs at late grain filling stage grown in the field in year 2019. Pictures of the representative plant senescence phenotypes of NILs and their parental varieties were taken just before harvest. **a**, IR72 (*indica*) and IR72-NIL. **b**, Milyang21 (M21; *indica*) and M21-NIL. **c**, Milyang23 (M23; *indica*) and M23-NIL. **d**, Junam (JN; *japonica*) and JN-NIL. **e**, Actual grain yields of NILs grown in the field. Data are means ± SE (n = 3). *$P < 0.05$ by Student’s t-test. Source data of Supplementary Figure 18e are provided as a Source Data file.

Supplementary Tables

Supplementary Table 1: QTLs for leaf senescence between *japonica* and *indica* subspecies

Population	Trait	Chr.	Distance (cM)	Physical position (Mbp)	Interval marker	LOD*	PVE[1] (%)	Add[2]	Dom[3]
IR72/Junam F2:3	Flag leaf	1	253.8-257.2	31.35-31.72	Chr01-216 - Chr01-224	7.2	12.6	35.2	6.7
		4	434.7-442.1	31.35-32.66	Chr04-148 - Chr04-153	7	11.7	-30.8	4.3
		8	102.0-103.6	6.25-8.31	Chr08-87 - Chr08-93	5	8.2	-30.2	0.1
		9	141.3-147.5	20.22-20.89	Chr09-99 - Chr09-107	13.1	24.2	49.1	25.5
	Second leaf	1	253.8-257.2	31.35-31.72	Chr01-216 - Chr01-224	9	15.8	37.1	-3.1
		4	434.7-442.1	31.35-32.66	Chr04-148 - Chr04-153	4.8	7.8	-21.8	8.3
		6	43.4-48.3	3.68-4.33	Chr06-20 - Chr06-24	4.6	7.7	26.5	2.9
		8	87.9-98.6	5.27-6.08	Chr08-80 - Chr08-81	5.2	8.5	-18.3	-17.8
		9	141.3-147.5	20.22-20.89	Chr09-99 - Chr09-107	12.7	23.9	45.4	2.6

*: LOD (logarithm of odds.) threshold value: 4.3

[1]PVE : Percentage of the phenotypic variance explained by each QTL.

[2]Add: Additive effect of the Junam allele on chlolophyll content at 45 days after heading.

[3]Dom: Dominance effect of the Junam allele on chlolophyll content at 45 days after heading.

Supplementary Table 2: Primers used for fine mapping

Name	Forward primer (5’→3’)	Reverse primer (5’→3’)	SNP specific primer (5’→3’)
RM24597	AACTCAGCCTCTCTGTCCTCTTCC	CCAGGGTGCAATTGTAGTGAGC	
RM24615	ATAGTTGACTGCACATGAGAGC	AACAGTCCAACTCCTCTTTGG	
RM24643	CCGACGCTGGTAAGCAAAGC	CCACTCGAAACATACCGAAACG	
RM24648	GACTCATCATGGCATCAATGTGG	AAGTACACTCTGCGCAAGATTTGG	
RM24661	CACAAACCGGCAATCTCTCTCC	GTGATCATGGCAAATCACTGACC	
RM24666	CTAGTGCACACGGGCGACAAGC	CTCCAGATGAGACGAGACCACACG	
RM24674	GTGTGGTTTCCTGCAACTTGAACC	TGGTCTCACCTCTCATGCATAGCC	
C9-1	TGTACCCTGAAATGAATCTCCCT	ACATGCTTTCCTCTTGCAGTT	
C9-2	AGAGCGCATACACCTCGGAT	CGACTCTCCGCCTCTCTCTC	
C9-3	ATCGGCTGCACTGTAACTGC	TGCTCGGTCAATTCATGCCC	
C9-4	TACACGCCAAAGGAACCTCC	AGTTCAGTCCTGTCAAAGAGAACT	
C9-5	GCAAGTTGCAACACCAAGAA	AGGAGAGCTCCATCGTTGCT	
C9-6	TCCCGATGACAAGTTGAGTGA	AGAGAGATGCTAAGTAACGGTATCT	
C9-7*	GACAGTGGATATATATTTTCGCACGCGAT	TGGATTTGTCTACATCAGGTCTGGTTCG	GTTCTTGTAGATGTTGCACATGCCACA<br>CTGTCTGAATATCGGACATATCACCCCC
C9-8	CTGCTATGGATTGGGTCGAGG	CACCTGCTTCTTCCGTCTCAA	
C9-9	GAGGCAAGCACTGTGGAGAG	TCATTATTGGGGCCCGGACT	
C9-10	TCTGTTTCCTGCAAGCAAGCT	GCTAGGGCGCACAAGTGAAT	
C9-11*	CAGTGTACTCAACATTTCAAGTAACTCG	GAGGATTCAGTCAAAAATATGATCACTC	CAAACACGATTCCAACTTCTTTTTTATA<br>CATGTAATTACACTTGGAATCTAGTTCAAA
C9-12	ATCTGCAGCCCACTACCCTT	TGGGTTGTTTCGGAAGCGAG	

C9-13	TGTTTGGCAGAGCTCCAACTTT	AGCTAGTTGGAGCACTTTCACA	
C9-14	TGGCTACATGGGCATTGAGA	TGCTCCTTGTTTTCGGTGGG	
C9-15	TCGATGTGACGTTTTTGGGAA	TCTGGACAAAGTACCTGCGT	
C9-16	CTCCACACGTCGCTCTTCAC	TGGCTTTTGAACGGTTCGCT	
C9-17	GGTGGCATTGGGTGTGACAT	CTCCTCTGACCCGGTCCATT	
C9-18	GCACTCCCGTCACTTTTTCCA	ACTGTTGTTGCTTGCTCCGT	
C9-19	TCTTATTAGTAGCGCGCGGT	TGTGTGTGAAATATAGTCGCATCT	
C9-20	AAGGAGGGTTGGGGGTGAAA	AACTCCCCAAGGGCTTCAGT	
C9-21	AAAGCGACACGACGACTTCC	GTCGAAAAGTCAAAGGCGTTA	
C9-22	TTGCATCCGAGTGTTCAGGC	CGTCGATTTCAGCCTCCTCG	

* Tetra-primer ARMS-PCR.

Supplementary Table 3: *Indica* and *japonica* varieties used in this study

Supplementary Table 3a. Cultivars used for generation of NILs

Supspecies	Variety	Haplo-type	Polymorphic promoter sites																				
			-1904	-1897	-1896	-1874	-1863	-1854	-1825	-1731	-1766	-1377	-1362	-1322	-862	-510	-443	-440	-414	-402	-397	-359	-236
Indica	IR72	1	A	T	T	A	C	T	A	G	T	AAAAGCTC	A	G	A	T	C	-	G	G	G	A	C
Indica	Milyanng21	1	A	T	T	A	C	T	A	G	T	AAAAGCTC	A	G	A	T	C	-	G	G	G	A	C
Indica	Milyanng23	1	A	T	T	A	C	T	A	G	T	AAAAGCTC	A	G	A	T	C	-	G	G	G	A	C
Japonica	Junam	8	G	A	A	G	C	C	G	G	G	--------	G	A	T	C	C	A	G	A	G	-	C
Japonica	Saeilmi	8	G	A	A	G	C	C	G	G	G	--------	G	A	T	C	C	A	G	A	G	-	C

Supplementary Table 3b. Agronomic traits of NILs grown in fields (*n* = 17)

Traits	Plant height (cm)	Panicle length (cm)	Panicle number per plant (n)	Spikelet number per panicle (n)	1,000-grain weight (g)	Grain yield (g/plant)	Grain filling rate (%)
IR72	63.5 ± 2.7	25.2 ± 0.6	16.3 ± 1.6	131.1 ± 8.2	23.6 ± 0.2	35.9 ± 4.8	68.8 ± 3.0
IR72-NIL	67.2 ± 2.8	25.9 ± 0.9	16.6 ± 1.5	132.1 ± 7.6	23.9 ± 0.5	39.6 ± 3.9	72.4 ± 3.0
P value	2.69E-06	1.45E-03	0.599	0.689	2.54E-02	0.017	1.02E-03
M21	73.0 ± 4.1	23.0 ± 2.6	13.6 ± 2.1	154.0 ± 11.5	25.1 ± 1.1	29.0 ± 4.5	69.6 ± 5.4
M21-NIL	73.1 ± 3.4	25.7 ± 1.2	13.8 ± 2.0	152.1 ± 11.0	25.7 ± 0.8	32.7 ± 4.2	76.3 ± 3.1
P value	0.925	7.66E-03	0.704	0.597	0.033	0.010	2.53E-05

M23	76.1 ± 4.0	23.5 ± 1.7	10.4 ± 2.70	142.9 ± 9.5	24.7 ± 0.5	29.1 ± 4.9	68.6 ± 5.4
M23-NIL	77.7 ± 4.1	24.0 ± 1.4	10.9 ± 2.5	137.8 ± 11.6	25.5 ± 0.4	32.6 ± 2.5	77.0 ± 7.4
P value	0.377	0.474	0.599	0.174	9.34E-06	0.013	8.08E-04
Junam	64.2 ± 1.6	21.1 ± 0.7	11.8 ± 2.2	105.5 ± 7.8	26.3 ± 0.4	30.7 ± 3.1	85.2 ± 3.0
Junam-NIL	70.4 ± 2.2	22.5 ± 1.1	13.1 ± 1.9	101.6 ± 9.1	24.4 ± 0.4	27.6 ± 4.8	78.7 ± 2.6
P value	4.77E-17	1.22E-07	0.049	0.154	9.29E-18	0.019	6.30E-09

Supplementary Table 4: Summary of SNP markers in the IR72/Junam F23 population.

Chr. No.	No. of markers	Length (cM)	Avr. of marker interval (cM)
Chr. 1	339	464.2	1.37
Chr. 2	168	318.0	1.89
Chr. 3	354	743.6	2.10
Chr. 4	170	549.2	3.23
Chr. 5	218	366.9	1.68
Chr. 6	264	471.9	1.79
Chr. 7	244	314.2	1.29
Chr. 8	249	502.5	2.02
Chr. 9	123	174.3	1.42
Chr. 10	272	391.8	1.44
Chr. 11	379	659.7	1.74
Chr. 12	272	427.4	1.57
Total	3,030	5,384	1.80

Supplementary Table 5: List of primers used for qRT-PCR, DNA constructs and genotyping in this study.

Name	Primer sequence (5′-> 3′)	Remarks
**Primers used for qRT-PCR**		
OsPRR95-QF	ACATGACGCATGCAAGAATATC	
OsPRR95-QR	CTTCCGTATTGGCTTAACAAGG	
OsSGR-QF	ATGCAATGTCGCCAAATGAC	
OsSGR-QR	GTTTCCATTTGCTTGCCATCT	
OsNAP-QR	TTGGTGCAACTTTCCAAATAGG	
OsNAP-QR	ATTCGCCATGTGCAATTATGTT	
OsUbi-QF	CTGCTGCTGTTCTAGGGTTCAC	
OsUbi-QR	CAAAACGTTTCAGACACCATCA	
**Primers used to generate DNA constructs**		
OsSGR-OXF	AAGCTTACTAAGAGATCCGAGGGAGCAG	Overexpression
OsSGR-OXR	GGTACCAACCAAACGAATTCGCTAATCTA	
OsSGR-CASF	GGCAGAGGGCGAGGCTGTTCGGGC	CRISPR/CAS9
OsSGR-CASR	AAACGCCCGAACAGCCTCGCCCTC	
OsSGR-RNAi-cF	GTACAAAAAAGCAGGCTCGCCATACAGCCTCATCC	RNAi
OsSGR-RNAi-cR	GTACAAGAAAGCTGGGTGCTGAGCTAAATGCCACTACG	
OsSGR-pro-F1	CACCGAGGAAGAGAGGGGATGGG	Protoplast-Promoter
OsSGR-pro-R1	GTCTGCTCCCTCGGATCTC	
**Primes used to generate wheat germ protein expression system constructs**		
OsSGR-WG-F	ACCGCGCGATAAGCTACCACCATGGCGAGGCTGTTCGGGCCGGC	
OsSGR-WG-R	CTTTGTAGTCAAGCTCCTGCTGCGGCTGGCCGTCG	
**Primers used for genotyping**		
3A-01206RS	TGCCACGTAGTACTGAAACCAC	Specific primers for *OsSGR-D1*
3A-01206L	TCAGAGTGATCGTTCCAAACAC	

RB	CAAGTTAGTCATGTAATTAGCCAC	T-DNA specific (right border)
3A-00334RS	AGTCCCCATATATTGCATGGTC	Specific primers for *OsSGR-D2*
3A-00334L	GAGAAGAAAGAGGATGGTGCAT	
5A-00143RS	GATGGACCAAACATATGGCC	Specific primers for *OsPRR95-D1*
5A-00143L	AGTATATGTGGCCAGCAGCC	
3A-13152LS	TTTTGCCTCCTAGTTGGGTG	Specific primers for *OsPRR95-D2*
3A-13152R	TAACTTGGGAGGGCAAAATG	
LB	ACGTCCGCAATGTGTTATTAA	T-DNA specific (left border)
**Primers used to isolate *OsSGR* cDNA for tobacco infiltration**		
OsSGR-NcoI	AACCATGGCTGCTGCTACTTCGACCATGT	
OsSGR-SpeI	AAACTAGTCTGCTGCGGCTGGCCGTCGGC	
**Primers used to amplification for *OsSGR* genomic region**		
OsSGR-p1	CAGCAAGGGTAGAGAGGAGAGA	Promoter region
OsSGR-p2	AGACTTGGCTCGTGGTTATCAT	
OsSGR-p3	ATGATAACCACGAGCCAAGTCT	
OsSGR-p4	GAGAGAGCGGGTTAAGTGAGAA	
OsSGR-c1	TTCTCACTTAACCCGCTCTCTC	Coding region
OsSGR-c2	TGTTGGAGATCAGTTTTTGGTG	
OsSGR-c3	ACCAAAAACTGATCTCCAACA	
OsSGR-c4	TTGCCATTTTTGCAGTAACATC	
