# Supplementary material for: Natural variations at the Stay-Green gene promoter control lifespan and yield in rice cultivars
Source: Nat Commun. 2020 Jun 4;11:2819. doi: 10.1038/s41467-020-16573-2 (PMC7272468; doi:10.1038/s41467-020-16573-2)
Supplement: Supplementary file 3 — Description of Additional Supplementary Files [file 41467_2020_16573_MOESM3_ESM.docx]

**Description of Additional Supplementary Files**

**File name:** Supplementary Data 1

**Description:** Polymorphisms and chlorophyll content of Accessions used in this study

**File name:** Supplementary Data 2

**Description:** Comparison of 101 SNP calls by GBS
